# Supplementary material for: The Effects of the Coating and Aging of Biodegradable Polylactic Acid Membranes on In Vitro Primary Human Retinal Pigment Epithelium Cells
Source: Biomedicines. 2024 Apr 26;12(5):966. doi: 10.3390/biomedicines12050966 (PMC11117638; doi:10.3390/biomedicines12050966)
Supplement: Supplementary file 1 [file biomedicines-12-00966-s001.zip › biomedicines-2928087-supplementary.pdf]

SUPPLEMENTARY INFORMATION

The Effects of the Coating and Aging of Biodegradable Polylactic Acid Membranes on In Vitro Primary Human Retinal Pigment Epithelium Cells

Georgina Faura [1,2,*], Hana Studenovska [3], David Sekac [4,5], Zdenka Ellederova [4], Goran Petrovski [6,7,8,9] and Lars Eide [1,10,*]

1 Department of Medical Biochemistry, Institute of Clinical Medicine, Faculty of Medicine, University of Oslo, 0372 Oslo, Norway
2 CIDETEC, Basque Research and Technology Alliance (BRTA), 20014 Donostia-San Sebastián, Spain
3 Institute of Macromolecular Chemistry, Academy of Sciences of the Czech Republic,
162 00 Prague, Czech Republic; studenovska@imc.cas.cz
4 Institute of Animal Physiology and Genetics, Academy of Sciences of the Czech Republic,
277 21 Libechov, Czech Republic; sekac@iapg.cas.cz (D.S.); ellederova@iapg.cas.cz (Z.E.)
5 Department of Cell Biology, Faculty of Science, Charles University, 128 00 Prague, Czech Republic
6 Center for Eye Research and Innovative Diagnostics, Department of Ophthalmology, Oslo University
Hospital and Institute for Clinical Medicine, University of Oslo, 0424 Oslo, Norway;
goran.petrovski@medisin.uio.no
7 Norwegian Center for Stem Cell Research, Oslo University Hospital, 0424 Oslo, Norway
8 Department of Ophthalmology, University Hospital Centre, University of Split School of Medicine,
21000 Split, Croatia
9 UKLO Network, University St. Kliment Ohridski, 7000 Bitola, North Macedonia
10 Department of Medical Biochemistry, Oslo University Hospital, 0424 Oslo, Norway
* Correspondence: gfaura@cidetec.es (G.F.); lars.eide@medisin.uio.no (L.E.)

**Table S1.** Primers used for DNA damage and mtDNA-CN.		
**Primers**		
	**Forward**	**Reverse**
**12S**	*5′-AAA CTG CTG CTC GCC AGA-3′*	*5′-CAT GGG CTA CAC CTT GAC CT-3′*
**NDUFA9**	*5′-GCA AGG GTC CCT ATG AGA GAA-3′*	*5′-CAA GAA CGA GGG GAA AAG TG-3′*
**PCR program**		
**Cycles**	**Temperature (°C)**	**Time**
1x	65	15 min
1x	94	10 min
40x	94	10 s
	60	1 min

**Table S2.** Primers used for gene expression.		
	**Forward**	**Reverse**
**BEST1 [14]**	*5′-GAATTTGCAGGTGTCCCTGT-3′*	*5′-ATCCTCCTCGTCCTCCTG AT-3′*
**RPE65 [25]**	*5′-GCCCAGGAGCAGGACAAAAG-3′*	*5′-GCGCATCTGCAAGTTAAAACCA-3′*
**PAX6 [25]**	*5′-AACGACACAGCCCTCACAAACA-3′*	*5′-CGGGAACTTGAACTGGAACTGAC-3′*
**ZO-1 (TJP1) [49]**	*5′-CCAGAATCTCGGAAAAGTGC-3′*	*5′-ACCGTGTAATGGCAGACTCC-3′*
**SOX9 [50]**	*5′-GTA CCC GCA CTT GCA CAA C-3′*	*5′-TCT CGC TCT CGT TCA GAA GTC-3′*

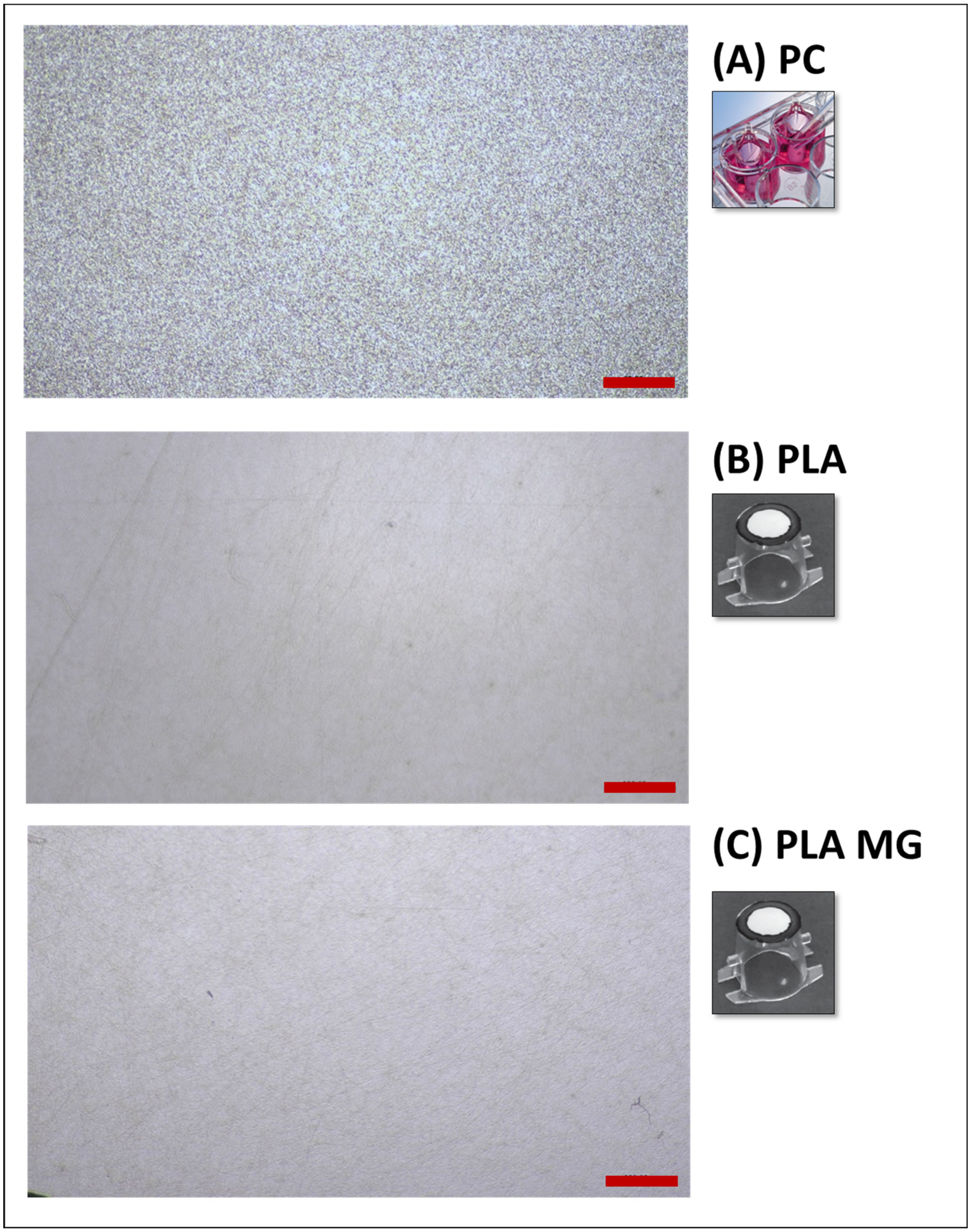

Figure S1. Representative optical microscope images of (A) commercial PC, (B) uncoated electrospun PLA membranes, and (C) coated electrospun PLA membranes without cells for comparison. Red scale: 300 μm. PC: polycarbonate; PLA: polylactide; MG: Matrigel.

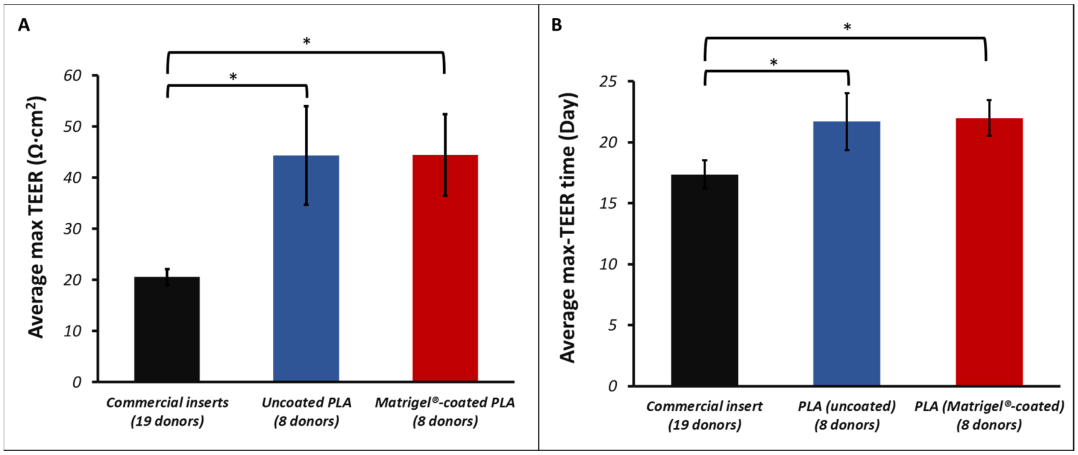

Figure S2. (A) Peak (max) TEER values and (B) peak TEER value per day. Average from all TEER monitoring sessions for each support type. PC: polycarbonate, PLA: polylactide. Error bars: SEM. * $p \leq 0.05$.

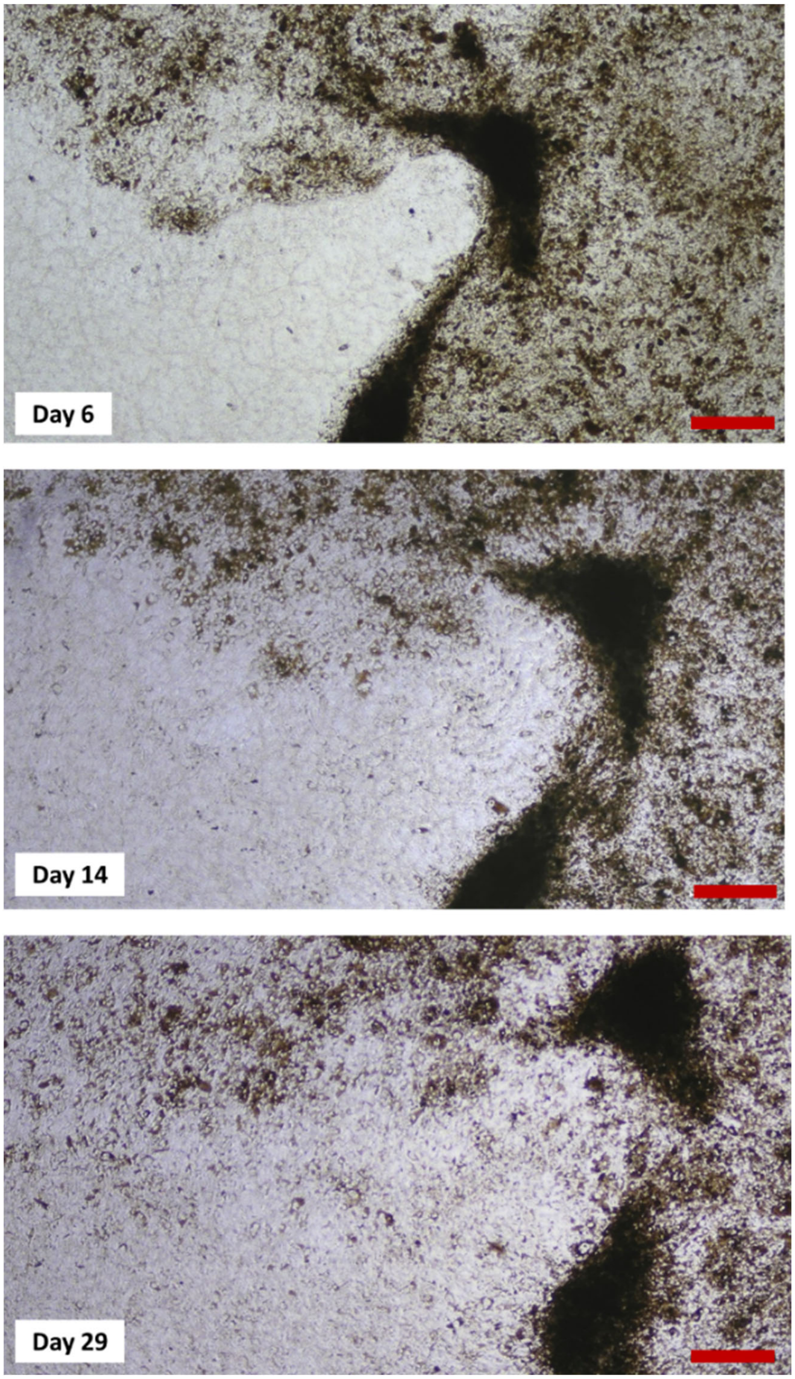

Figure S3. Representative image sequence of hRPE cell culture on uncoated PLA. Red scale: 300 μm.

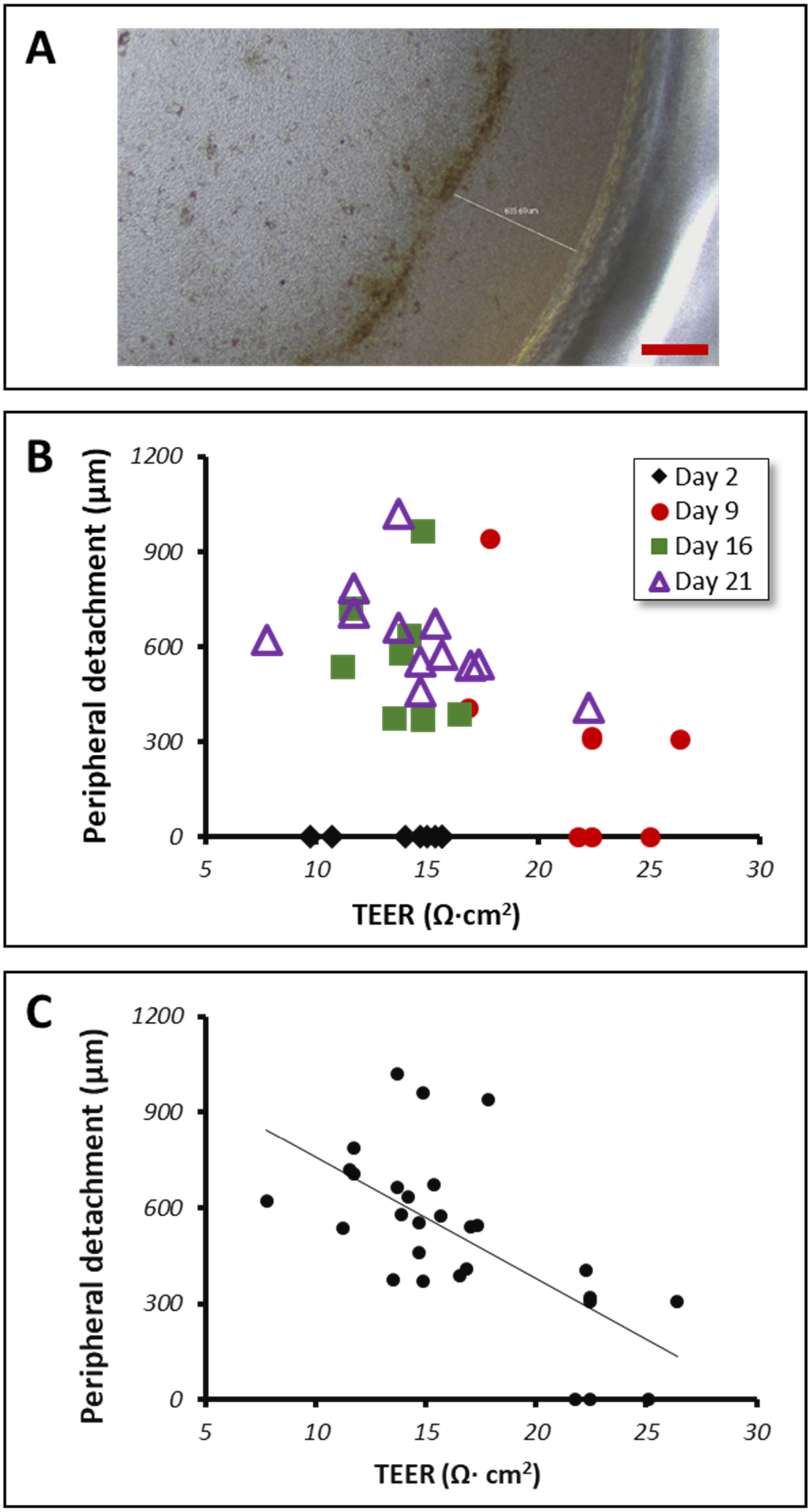

Figure S4. (A) Optical microscope image of hRPE cell culture on commercial insert and measurement of peripheral partial detachment. Red scale: 300 µm. (B) Relation between detachment and TEER at diverse culture days and (C) all days merged (excluding day 2).

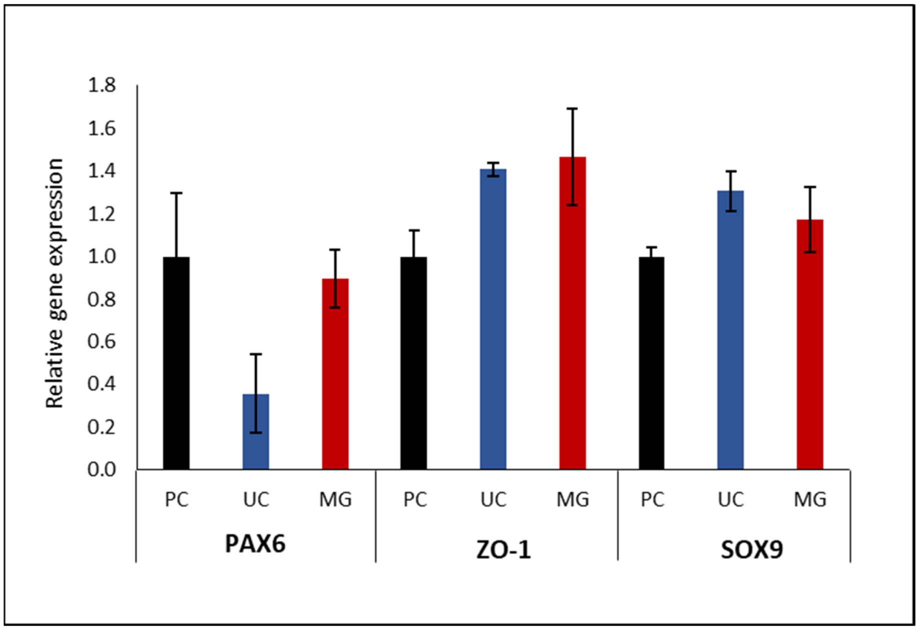

Figure S5. Relative expression of hRPE markers; PAX6, ZO-1, and SOX9 in hRPE cultivated on uncoated and coated PLA membranes in comparison to commercial polycarbonic inserts. Expression of monitored genes in commercial inserts was set to 1. Data are shown as ratio. PC: polycarbonate, UC: uncoated PLA membranes, MG: coated PLA membranes; 3 donors; error: SEM

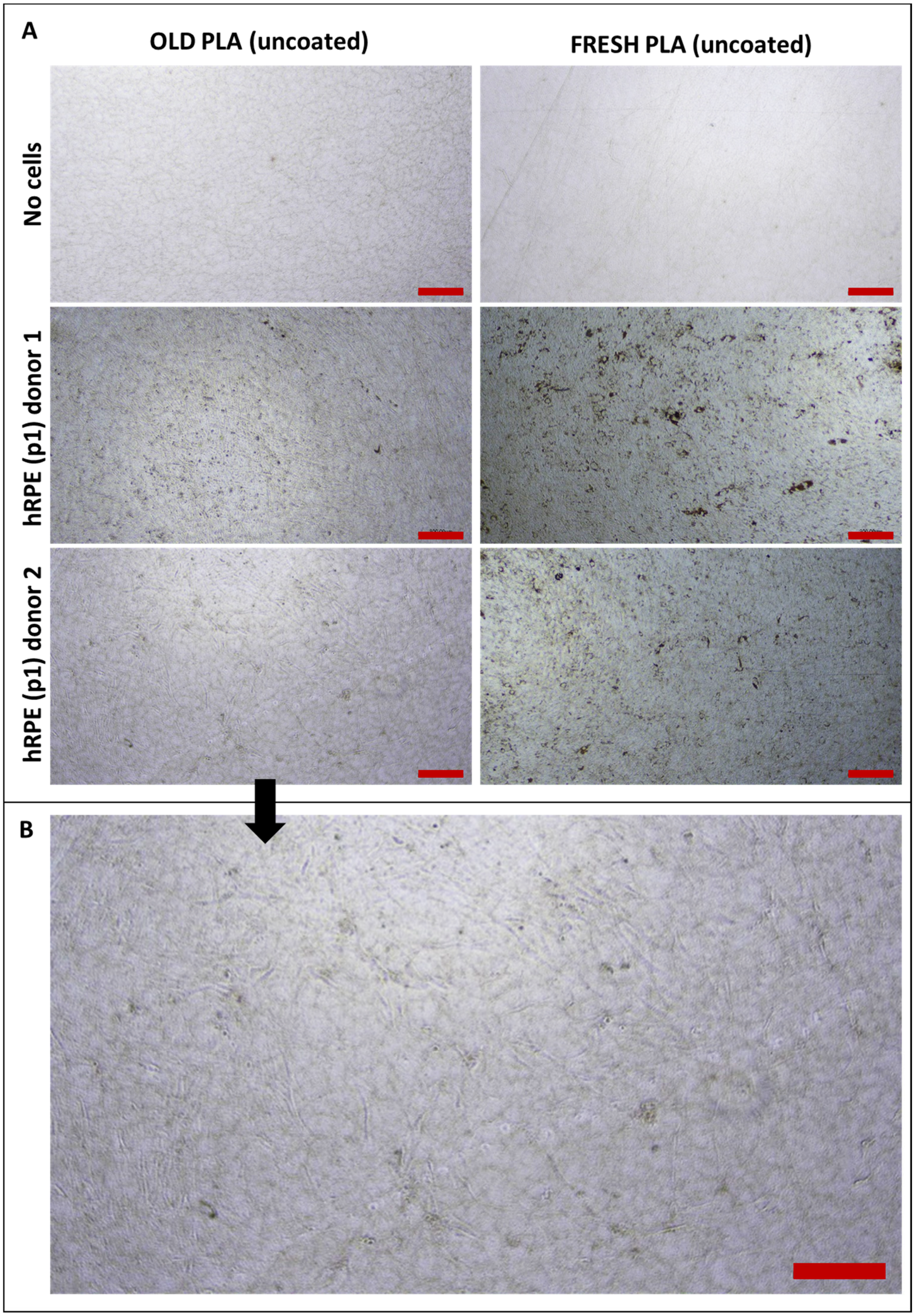

Figure S6. Representative images (20th day of culture) (A) comparing hRPE (passage 1) morphology on old (21 months) and fresh (1 month) uncoated PLA. Pictures of coatings without cells are also shown for reference. (B) Close-up image of hRPE culture (donor 2) on uncoated old PLA. Red scale: 300 μm.

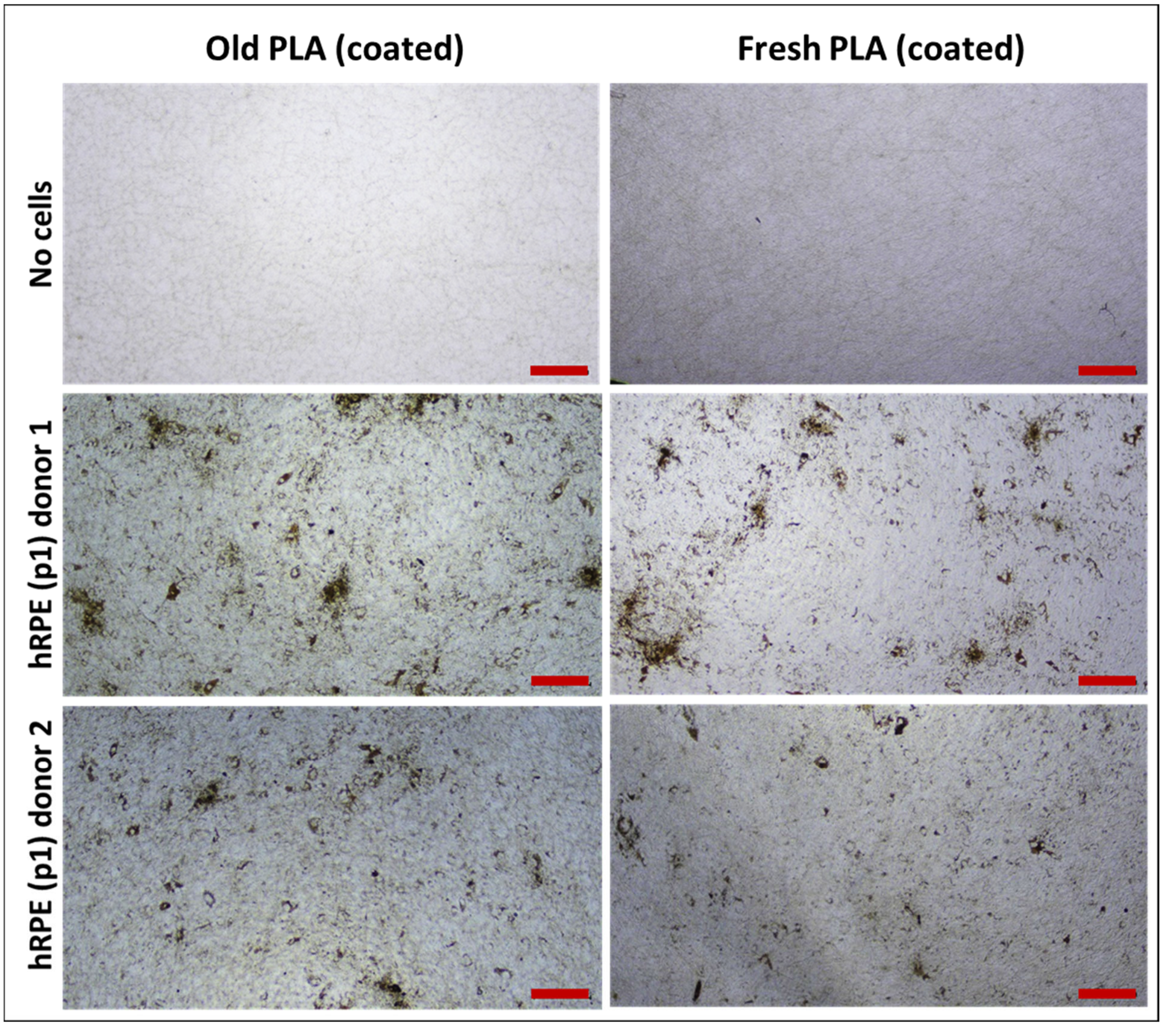

Figure S7. Representative images (20th day of culture) comparing hRPE (passage 1) morphology on old (21 months) and fresh (1 month) PLA coated with Matrigel®. Pictures of coatings without cells are also shown for reference. Red scale: 300 µm.

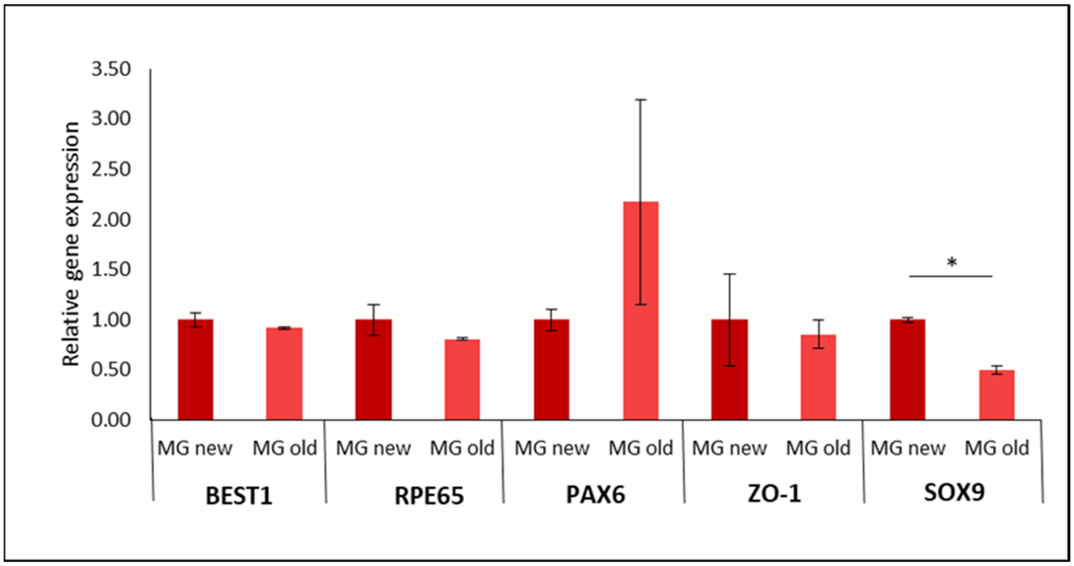

Figure S8. Relative gene expressions of BEST1, RPE65, PAX6, ZO-1, and SOX9**;** 2 donors; error: SEM. * $p \leq 0.05$
